# Five advanced pancreatic cancer patients in a Phase I study of anti-CD3 x anti-EGFR bispecific antibody armed activated T cells (BATS)

**DOI:** 10.1186/2051-1426-3-S2-P55

**Published:** 2015-11-04

**Authors:** Lawrence G Lum, Minsig Choi, Archana Thakur, Abhinav Deol, Kristie Fields, Elyse Tomaszewski, Dana Schalk, Vidya Kondadasule, Greg Dyson, Hemchandra Mahaseth, Philip Philip, Anthony Shields

**Affiliations:** 1 Wayne State University & Karmanos Cancer Institute, Detroit, MI, USA; 2 Stony Brook University School of Medicine, Stony Brook, NY, USA

## Background

Conventional chemotherapy for locally advanced pancreatic cancer (LAPC) and metastatic PC (MPC) is associated with clinical toxicities, dismal response, and poor survival rates. Novel approaches are needed. Preclinical studies show that bispecific antibody armed T cells exhibit cytotoxicity, proliferate, and secrete cytokines(CCR 12:569, 2006) and that anti-CD3 x anti-EGFR Bispecific antibody Armed T cells (EGFR BATs) can target and kill EGFR+ colon and pancreatic cancer cells (CCR 12: 183, 2006). We conducted a Phase I using EGFR BATs in LAPC, MPC and colorectal cancer to determine safety, feasibility, efficacy, and immune responses.

## Methods

Peripheral blood mononuclear cells from leukapheresis were stimulated with anti-CD3 and IL-2 to produce activated T cells that were armed with EGFRBi to create EGFR BATs and cryopreserved. Patients with colorectal cancer (6), LAPC (3), and MPC (2) were treated with EGFR BATs after 1 cycle of FOLFOX6 (oxaliplatin and leucovorin, 5 FU) given to create immune space. The doses of BATs in this Phase I trial were 10, 20, and 40 x 10^9^ BATs/infusion given in 3 weekly infusions with a booster infusion at 3 months.

## Results

We report the data on pancreatic cancer patients in the study. One patient failed to grow activated T cells. Of 5 PC patients in the study, 4 received 4 infusions, and 1 received 3 infusions. There were no dose limiting toxicities with an average of 48 x 10^9^ BATs (9.3 -74 x 10^9^) infused. Grade 1-2 headaches, fevers, chills and blood pressure changes were observed. Two patients (IT20091 and IT20104) went into CR after restarting chemotherapy after “apparent” progression of disease. One patient (IT20102) had stable disease with near PR (↓27% of an index lesion) for 229 days. IT20104, who received 3 infusions, had a lymph node increased by >50%, was given chemotherapy, and went into CR after chemotherapy. Immune responses to PC lines were detected in the PBMC. Time to progression was 71, 138, 186, 211, and 229 days with a median time to progression of 186 days, and OS was 297, 346, 409, 436, and 677 days with a median OS 14.5 months.

## Conclusions

Stable disease, encouraging increased times to progression and survival times, and clinical responses after chemotherapy suggest that EGFR BATs infusions have clinical activity in PC. An interesting observation is that immunotherapy may be altering the tumor microenvironment or tumor to improve chemotherapy responses.

